# Preventive health services for young children in Israel: historical development and current challenges

**DOI:** 10.1186/s13584-019-0287-7

**Published:** 2019-02-07

**Authors:** Deena R. Zimmerman, Gina Verbov, Naomi Edelstein, Chen Stein-Zamir

**Affiliations:** 1 Jerusalem District Health Office - Ministry of Health, 86 Jaffa Road, 94341 Jerusalem, Israel; 2 The Hebrew University of Jerusalem, Faculty of Medicine, the Hebrew University and Hadassah Braun School of Public Health and Community Medicine, Ein Karem, PO Box 12272, 91120 Jerusalem, Israel

**Keywords:** Well child care, Preventive medicine, Children, Health care services

## Abstract

**Purpose:**

Pediatric preventive health services are delivered in many different formats throughout the world. In Israel, these services for young children are provided in designated Maternal Child Health Clinics (MCHC). The history and operation of Israel’s MCHC have been described primarily in the Hebrew language medical literature with most of these reports being over a decade old. This paper provides an up to date summary of the evolution and current care in Israel’s one-hundred-year old model for the provision of preschool preventive health services. As these clinics have been recognized by the World Organization as a model for emulation, it is important that such information be made available.

**Abstract body:**

Israel’s MCHC provide universal care to infants and preschool children (0–6 years), free of charge. These community-based clinics provide developmental surveillance, growth monitoring, and routine childhood immunizations. Anticipatory guidance is offered to families on topics including nutrition, parenting and child safety. Screening is also performed for maternal postpartum depression and family violence. Care is given by public health nurses working in collaboration with physicians.

The vast majority (> 96%) of the country’s children receive care in this system. Immunization coverage rates through this system are in line with World Health Organization guidelines – over 95% overall average nationally. Unfortunately, the allocated funding has not increased in proportion to the population growth. There is ongoing debate on the role of the national government in health services: should it be that of a direct services provider or focus on guidance and regulation of the health system.

**Conclusion:**

MCHC well child care can help assure widespread provision of pediatric preventive health care. For this model to function, both its funding and content needs to be updated on an ongoing basis to reflect current preventive health care needs.

## Background

Primary care is viewed as a cornerstone of health care [1] The provision of Israel’s primary care for children is divided into curative and preventive services. According to the National Health Insurance Law (NHIL, 1995) [2], curative health care is provided by one of four health funds among which Israeli residents can choose freely [3]. Health care is provided either in clinics run directly by the health funds or in “independent practices” whose services are contracted by the health funds [4]. Curative primary care pediatrics is provided by a combination of pediatricians and family practitioners, with younger children (by parental preference) more likely to be followed by pediatricians [5]. Children under the age of 18 years are entitled to health insurance regardless of their residency status [6].

Preventive care for young children (from birth to 6 years) is provided as a designated service via Maternal Child Health Clinics (MCHC) of which there are currently some 1000 community-based MCHC. These clinics are operated by the Ministry of Health (providing services to 64% of the children nationally, 2014 report), two municipalities (Jerusalem, 10.4% and Tel Aviv, 4.1%), the 4 health funds (21% of children) and other NGOs (0.5%) [7–10]. This well child care system has an over one-hundred-year history that predates the establishment of the State of Israel, yet its format and existence continues to be debated. The goal of this paper is to describe the evolution, current status, and possible development of preventive health services for children in Israel.

## Evolution of the MCHC

Infant mortality rates at the beginning of the twentieth century in the population residing in the region of what is currently Israel were very high. The primary causes were poor sanitary conditions, poor nutrition, infectious diseases and the lack of a cohesive medical infrastructure. Additional social conditions, such as the immigration of young families without the older generation, who had traditionally guided new parents through the early stages of parenthood, also contributed. The change began with the visit of Henrietta Szold, an American social activist in 1909–10. After seeing the poor sanitary conditions and learning of the high infant mortality, she decided to focus on preventive medicine. With the help of philanthropist Nathan Strauss, a service similar to the Henry Street Settlement in New York City, was founded in Jerusalem in 1913. Its work was curtailed during World War I and reopened in 1918. At this point, it was named “Tipat Chalav”, the Hebrew version of the French name for similar clinics “Gout de Lait” (drop of milk). Through efforts of the Hadassah Organization, Women’s’ International Zionist Organization, and the Clalit Health Fund, the number of clinics increased to over a hundred in 1948. With the establishment of the state, responsibility for the MCHC was handed over to the nascent national government, with care of all providers delivered according to the Ministry of Health directives [11]. In 1995, the right to universal health care was mandated in the NHIL. This was the first time that the health basket of preventive pediatric health services (MCHC and school health services) was anchored in law.

According to the 1995 NHIL, the MCHC services were scheduled for transfer to the health funds within 3 years. This was part of an overall plan to reduce direct provision of services by the government in many domains and have the ministry of health function mainly as a regulator, as suggested by appointed commissions [12, 13], as well as diminishing the governmental workforce. Towards this goal, a pilot program initiated in 2004, in which responsibility for the MCHC services in selected localities would be transferred from the Ministry of Health and municipalities to the health funds, with special governmental funding for this purpose. After extended negotiations among the parties (the Ministry of Health, the Ministry of Finance, the health funds and the municipalities), the scope of the pilot was reduced from the originally planned 40 localities to three (Tel Aviv-Jaffa, Modi’in and Elad). In 2007, political opposition to the pilot intensified with the National Council for the Child, the Adva Centre (a non-governmental organization addressing inequalities), the Israel Medical Association together with the public health nurses and their union. In February 2007, the Prime Minister decided to halt the pilot and several months later, that decision was confirmed by the Government.

Despite a yet another committee commissioned by the Ministry of Health in 2016, the current situation is still a conglomerate of providers with a maintenance of a fairly consistent distribution.

### Achievements

The MCHC system have recorded many achievements. By the early 1950s, MCHC care enabled improvement in child health status with decrease of infant mortality rates similar to developed countries [11]. Palti et al. described in the 1980s a program carried out by the public health team in the MCHC using a model of integration of early infant stimulation. The long-term evaluation of the program revealed positive effects on child development [14]. This model is now in widespread use, adding a developmental promotive aspect of childcare to pediatric health care, which had previously focused on physical health and acute situations. Similarly, a targeted breastfeeding promotion program carried out in the MCHC was effective in increasing the percentage of mothers breast-feeding fully or partially [15].

The existence of such an organized pediatric preventive health system has received international recognition and accolades. In 1998, the WHO presented Israel with a certificate of achievement “worthy of national recognition” for a “national community-based children’s health project that promotes ‘health for all’ values of equity, solidarity, participation, inter-sectoral approaches and partnership” [16].

#### Utilization

MCHC have been documented as being widely used by the population. In 2004, Palti et al. reported that the MCHC services for children in Israel are used by 96% of Jewish mothers and 100% of Arab mothers. The authors concluded that the preventive services provided by the MCHC contributed significantly to the continuous improvement of health indicators of mothers and children in Israel [17]. In 2007, a survey of 2575 mothers conducted by Rosen et al. reported a 95% immunization coverage rate and that 95% of children had been seen at least once by a MCHC physician [18]. In 2011, Gofin and Gofin reported population coverage in these centers of close to 100% [19]. Data from the National Quality Indicators Program show a high rate of regular utilization of early MCHC services, as 91% of children had three measurements of head circumference in the first 8 months of life [20].

##### Services

The current timing of recommended MCHC pediatric visits by MOH directives is outlined in Table 1 [21]. Each of these visits follows a clear format including determining parental concerns, measuring and plotting the child’s growth, structured developmental assessment and anticipatory guidance regarding nutrition, child development and family dynamics. Routine childhood vaccinations are administered according to the national standardized schedule (Table 2) and vision screening is scheduled at ages 3 and 5 years. It should be pointed out, however, that certain activities documented in the 2004 directive are no longer relevant. Since the advent of universal hearing screening at birth in 2010, behavioral hearing screening at age 7–8 months has been discontinued [22]. Formal communication screening at age 2 years has been replaced by inclusion of communication related items in the developmental surveillance.

**Table 1:** MCHC Services by Age

	Age	Parental guidance	Immunizations	Vision screen	Development evaluation	Physician exam	Growth
1	Birth - 2 weeks	X			X		HT, WT, HC
2	4–6 weeks	X	1 month		X		HT, WT, HC
3	8–10 weeks	X	2 months		X	2 months	HT, WT, HC
4	18–20 weeks	X	4 months		X		HT, WT, HC
5	26–28 weeks	X	6 months		X		HT, WT, HC
6	31–40 weeks	X			X	9 months	HT, WT, HC
7	48–58 weeks	X	12 months		X		HT, WT, HC
8	18–19 months	X	18 months		X	1.5 years	HT, WT, HC
9	24–30 months	X	24 months		X	2.5 years	HT, WT
10	36–42 months	X		X	X		HT, WT
11	60–72 months	X		X	X	5.5 years	HT, WT

*HT* height /length, *WT* weight, *HC* head circumference

**Table 2:** Routine MCHC Childhood Vaccinations

Age in Months	HBV	DaPT-IPV-HIB	bOPV	Rotavirus	MMRV	PCV 13	HAV
0	x						
1	x						
2		x		x		x	
4		x		x		x	
6	x	x	x	x			
12		x			x	x	
18			x				x
24							x

*DTaP-IPV-HIB* diphteria, tetanus, acellular pertussis, inactivated polio, hemophilus influenza B vaccine, *bOPV* bivalent oral polio vaccine, *MMRV* measles, mumps, rubella, varicella vaccine, *PCV 13* pneumococcus 13 valent vaccine, *HBV* hepatitis B vaccine, *HAV* hepatitis A vaccine

While the overall directive has not been updated in over a decade, many guidelines for specific services have been. Nutrition guidelines for children were updated in 2012. Major changes included removing age restrictions for introduction of specific solid foods after 6 months of age (except for honey and cow’s milk) [23]. The guidelines regarding anemia prevention were updated in the same year and encouraged the continuation of supplementary iron drops to 18 months rather than one year of age [24]. The guidelines for growth and nutritional monitoring were updated in 2014 [25]. The developmental surveillance directive was last updated in 2016 [26]. Key changes were assuring structured prompting of parental concerns at each visit and the addition of items that search for red flags for autistic spectrum disorder.

As indicted by the name, Maternal Child Health Centers include prenatal care for low risk pregnancies. [27] However, the increased technical nature and the medical approach to prenatal care have lead most women to seek these services via the health fund [10]. As a result, most MCHC no longer provide obstetric services (except for peripheral areas) [28].

MCHC pediatric services are currently provided uniformly to all children free of charge. In a country suffering from much healthcare inequity [29], this standardized and universal care provided by MCHC can serve as a model for improving such disparities. All MCHC visits are documented in electronic medical records. All government MCHC and the MCHC of one health fund use a uniform web-based computerized program since 2015. The three other health funds document the visits in the same proprietary computerized program that documents the acute care. The computerize nature of MCHC care and documentation enables service evaluation for purposes of program implementation, quality assurance and healthcare policy [30–32]. Use of MCHC records allowed for conducting case control studies regarding communicable diseases [33] and evaluation of the effectiveness of routine childhood vaccination programs [20].

The computerized documentation has also facilitated quality assurance. The National Program for Quality Improvement in Maternal Health Centers begun in 2015. One of the first topics for assessment was routine immunization timeliness. While overall immunization rates in Israel by age two years are high, 2016 EMR data showed that only 75% of children had completed 4 doses of DPT-IPV-HIB by 18 months of age and only 60% had received the MMR by 13 months of age (the minimum age of administration is 12 months) [20]. This corroborates a recent study of age-specific vaccinations assessment which showed considerable delay in receipt of routine vaccinations. While most (96, 95, 91, 96, 94 and 86%) children were vaccinated up-to-date for third HBV, fourth DTaP-IPV-Hib, third PCV13, first MMR/MMRV and both HAV doses; only 26, 29, 47, 64, 55 and 12% were vaccinated age-appropriate. Notably, the proportion of vaccinated children was 96% for third HBV scheduled at 6 months compared to 86% for second HAV scheduled at 24 months [34]. In the quality indicators report, the national government MCHC’s performance came in second overall with the highest being Maccabi health services. However, it is hard to compare providers as health fund clinics generally service less than 100 infants and those of the national government and municipalities service 500 to over 1000. Moreover, the characteristics of the population served by the governmental-municipal clinics differ from that served by health funds; children residing in low socio-economic localities (rank 1–3 of a 1–10 ascending scale) are served by the governmental-municipal clinics. However, it is hoped that the provision of provider specific statistics to each entity and publication of the findings to the general public will spur programs for improvement. Anecdotally, this is in fact happening as with the receipt of reports, outreach is done. The percentage of mothers screened for postpartum depression (77% nationally with target of 80%) is published as well. The baseline values for 2017 indicators (newborn follow up, breastfeeding, anemia, and domestic violence) newborn follow up, breastfeeding, anemia, and domestic violence have recently be released [20]. Anemia has also been chosen as a topic for which there are quality indicators (but results not yet published). Here too, it is hoped the monitoring of these topics will show the current level of implementation and allow the following of improvement trends.

In addition to the key role of the public health nurses in Israel in maintenance of nation-wide immunization coverage and addressing issues of childhood vaccinations. [35], MCHC play a crucial role in containment of infectious disease outbreaks. This is seen in both sporadic outbreaks of measles [33] and nation-wide project such as the re-institution of oral polio vaccine [36]. The current measles outbreak is providing yet another example of their important work.

##### MCHC staff

The main health care provider in the MCHC is the public health nurse. The MCHC physician is scheduled to provide five mandated visits (Table 1) and additional visits if indicated by the examination findings or professional assessment. However, only a single sentence in the directive describes the role of the physician.

In a previous comprehensive study, we explored the opinions of key stakeholders (MCHC physicians, non-MCHC physicians, MCHC nurses and parents) regarding the expected role of the MCHC physician [37]. These findings, and review of the literature of evidence based well childcare world-wide, led to the writing of a guide for the MCHC physician. This guide, a joint project of the Maternal Child divisions of the Jerusalem District Health Department and Ministry of Health, outlines the role of the physicians including the needed knowledge base and a visit-by-visit description of expected history, targeted physical and neurodevelopmental examination and anticipatory guidance. Those items listed are designed to complement, not repeat that performed by the nurses. The process has begun for writing a new updated set of directives, which will reflect the current needed role of the MCHC physician. Similarly, a study was recently completed that examined the need for possible update of the role of the MCHC nurse [38].

#### Current challenges

Despite important achievements to date, the system faces a number of challenges:

##### Sustainability

The lack of provision in the NHIL for updating the budget of the MCHC (capitation formula), has led to a lack of alignment of growth of the allocated budget with growth of the population. The number of live births in Israel increased considerably (over 33%) from 121,333 in 1996 to 183,314 in 2016 [39]. During this time period, the budgeting for MCHC personnel and infra-structure has not been increased in a matching pace and hence the ratio of children per health care worker has increased. The lack of approval of new positions impedes recruiting new nurses. In addition, the responsibilities of the MCHC nurses have steadily expanded due to medical and public health developments and the awareness of social problems further increasing the workload [40].

As shown the MCHC physicians study [37], there is concern about replacing MCHC physicians as a large part of the current cadre reaches retirement. However, the authors’ experience in the Jerusalem district has shown that it is possible to attract well trained, enthusiastic physicians from a number of medical specialties (pediatrics, family medicine and public health) for part time work along with working in curative health care settings. Recruitment efforts included attracting new immigrants from countries with a strong tradition of well child care as well as physicians post retirement. It is also possible to design a program for pediatric, family practice and public health residents that includes a structured curriculum with mentorship to help train the next generation of physicians. It is important that this experience be expanded nationwide.

##### Staff shortages

The lack of appropriate government funding prevents creating new professional positions to meet the needs of the growing population. It is also difficult to recruit nurses and physicians for the currently available positions, as 1) there is a national shortage of both nursing [41] and primary care physicians [42] and 2) the existing pay scale is markedly less than that offered in other health settings in Israel.

Staff shortages are felt in a number of areas. 1) There is a marked reduction of home visits. According to the directive, all first born children and all children at risk should have a home visit by two weeks of age. In most settings, due to staff shortages, this happens only for very high risk children if at all, 2) While all families who call for appointments for children up to age 6 will be given appointments, outreach efforts are often limited to children who are still at the ages of scheduled routine immunizations, 3) Physician checkup often only take place for the youngest children and 4) Telephones calls may not be answered when parents call, again due to staff shortages.

##### Structural uncertainty

As described above, the pros and cons of transfer of MCHC continue to be debated. There are a number of cogent arguments for and against transfer [43]. On a professional level, one key argument for transfer from separate government run clinics is to have both curative and preventive care under one roof. This would, allow the same primary care physician see the child in both sickness and in health. It would allow all the child’s medical information for be in one electronic medical record and would allow “one stop shopping.” Currently, if a child is found in the MCHC to have a condition requiring work-up (e.g. laboratory or radiological examinations), then the government MCHC staff refer the child to the health fund staff for further evaluation and treatment. Transfer of the MCHC service to the health funds would thus facilitate continuity of care and smooth access to the diagnostic and treatment facilities of the health fund. The counterargument is that health fund primary care clinics are already overburdened [44], and thus it is not clear that the child would actually be seen by the same physician, nor that the health fund would be able to provide the protected time for the staff to deal with well-child issues. Currently, health fund acute care physician visits are at best 10 min [41], which is far less than the 15–20 min allotted in MCHC. In a setting of limited professional resources, “the urgent would take precedence over the important”, that is, the health fund physician would feel impelled to first address the immediate needs of the acutely ill children, at the expense of preventive well- child care [43, 45]. An additional rationale for a separate MCHC staff is to have providers who are trained to focus on growth, development and behavior. At present, many health fund pediatricians have completed residency training in behavioral and developmental issues that are a major focus of well child care [46]. Health fund physicians also do not, in general, have continuing medical education that focuses on updates in Ministry of Health guidelines regarding well child care. Should the physician component be transferred to the health funds, it will be important that mandatory continuing medical education for MCHC physician staff be included.

Another professional concern regarding the supplier of MCHC services is that of infection control. All government MCHC are separate facilities that treat only well children. The need to separate well children from sick children either spatially or temporally has proven challenging for many health fund clinics.

On an economic level, it is contended that taking the government out of direct employment of MCHC staff would be financially beneficial. A similar argument was made for the privatization of the student health service, Israel’s preventive health care services for school age children. This has been shown to have negatively impacted the care given without any financial savings [47, 48]. While assigning preventive health services for children to the health funds is not privatization, there is no clear evidence that this would indeed save money.

Accessibility is yet another parameter to consider. Funding shortages have led to the combining of smaller MCHC with larger MCHC in the name of efficiency, but often reducing convenience to the parents. Lack of funding makes opening additional clinics in newly populated areas challenging, further leading to parental inconvenience. Health funds have multiple clinics which could, incorporate MCHC care. However, health fund clinics are not always evenly distributed. As the health funds compete to attract members, there may be multiple clinics in more attractive areas and few, if any, clinics in peripheral areas. Appointments tend to be more available in health fund clinics than in government MCHC. However, government MCHC have recently expedited appointment making by providing a centralized internet and call center.

There is also a concern that if the responsibilities within a locality are to be divided among Israel’s four competing health funds, none of them would have a community-wide perspective on health promotion needs and epidemiological events. This is in contradistinction to the government MCHC, which play a major part in community programs such as the Intergovernmental National Program for At-risk Children and Youth [49] and health promotion programs aiming to promote healthy nutrition and physical activity [32, 50]. Furthermore, as the government-based clinics serve all members of the local community, regardless of which health fund they belong to or their residential status, the staff of such clinics are more proficient in performing local community diagnosis and thus proffer community-oriented primary care. As pointed out by Freed et al., transfer of MCHC care could have a potential negative impact on Israel’s excellent immunization rates [51].

The prolonged uncertainly regarding the future provider of the MCHC services has inhibited the government in prioritizing the issue of sustainable funding for services provided by the clinics. The murkiness regarding the future has dampened enthusiasm and hampered the ability of the public health service to properly support MCHC care. There is hope that the current government focus on the first years of life may increase funding, however, this has not yet been implemented.

As it appears that current situation of multiple providers with a consistent distribution between them is going to continue for the forseeable future, it is important to work towards getting the maximum benefit. Towards this goal, there is a clear need for improvement in communication between the preventive and curative health services. Allowing for electronic communication and information sharing between providers would address this issue, while still providing the disease free setting and safeguarded time of a separate curative service. Interventions have been initiated such as a National Immunization Registry [31] to improve the continuity of care between the preventive and curative pediatric health systems. Another alternative is to consider a hybrid approach of leaving the nursing component of the MCHC in government hands (thus preserving the public health strength of the MCHC) but allowing for contracting of health fund physicians to provide the physician component in the MCHC clinics. Physician presence in MCHC clinics permits the interdisciplinary communication that provides optimal care. We believe that health fund physicians practicing in the same communities and treating the same children in sickness and in health would provide a much needed comprehensive medical home. By working under the aegis of the MCHC system, the pediatric healthcare that would be provided would be consistent and dependable.

Recently, a committee consisting of members of the Israel Pediatric Association, the Israel Family Medicine Association, the Ministry of Health and Goshen Organization (a pediatric organization dedicated to the promotion of community pediatrics) Well Child Care Clinical Guidelines approved by the Israel Medical Association. In addition to assuring continued uniform, evidence based care, it is hoped that this will be a first step in educating parents as to the importance of bringing children to MCHC for the full 6 years of care provided. At present, many parents currently feel that the sole purpose is to provide immunizations and thus the attendance rate drops off sharply after age 2.5 when the routine preschool immunizations are completed.

## Conclusions

The existence of Maternal Child Health Centers providesuniversal pediatric preventive health care in Israel. For this model to function effectively and efficiently, both its funding and content need to be ratified and updated on an ongoing basis to reflect current preventive health care needs. For improvement efforts to continue without fear of future instability it is essential that a clear structure, even if it is a composite of multiple providers, is outlined and stabilized.

## Abbreviations

DTaP- IPV- HIB: Diphteria, Tetanus, acellular Pertussis, Inactivated Polio, Hemophilus influenza B Vaccine
HAV: Hepatitis A Vaccine
HBV: Hepatitis B Vaccine
MCHC: Maternal Child Health Centers
MMR: Measles, Mumps, Rubella, Vaccine
MMRV: Measles, Mumps, Rubella, Varicella Vaccine
NHIL: National Health Insurance Law
OECD: Organization for Economic Cooperation and Development
PCV 13: Pneumococcus 13 Valent Vaccine
